# The Use of Mesenchymal Stromal/Stem Cells (MSC) for Periodontal and Peri-implant Regeneration: Scoping Review

**DOI:** 10.1590/0103-6440202406134

**Published:** 2024-10-25

**Authors:** Nidia C Castro dos Santos, Khalila C Cotrim, Gustavo L Achôa, Eduardo C Kalil, Alpdogan Kantarci, Daniela F Bueno

**Affiliations:** 1 Dental Research Division, Guarulhos University, Guarulhos, SP, Brazil; 2 School of Dental Medicine, Albert Einstein Israelite Hospital, São Paulo, SP, Brazil; 3 The ADA Forsyth Institute, Cambridge, MA, United States; 4 Núcleo de Pesquisa e Reabilitação de Lesões Lábio Palatais Prefeito Luiz Gomes, Oral and Maxillofacial Surgery Department, Joinville, SC, Brazil; 5 School of Dental Medicine, Harvard University, Boston, MA, United States

**Keywords:** Stem cells, stromal cells, peri-implant regeneration, intrabony defect, furcation

## Abstract

The necessity for regenerating peri-implant and periodontal tissues is increasingly apparent. Periodontal diseases can result in a significant loss of clinical attachment level, and tissue regeneration stands as the ultimate goal of periodontal therapy. With the rise of osseointegration, the prosthetic rehabilitation of missing teeth using dental implants has surged, leading to a frequent need for alveolar bone regeneration around implants. This review assessed studies reporting various sources of mesenchymal stromal/stem cells (MSC) and their potential in regenerating periodontal and peri-implant bone tissue. A search was conducted across seven databases spanning the past decade. Three authors independently screened all identified titles and abstracts for eligibility, generating tables to summarize included studies in animals and humans separately. A total of 55 articles were chosen for final evaluation, showcasing five origins of MSC used in humans and animals for regenerating periodontal tissues and peri-implant bone, using different types of scaffolds. Overall, research from the past decades supports the effectiveness of MSC in promoting periodontal and peri-implant regeneration. However, the impact of MSC on regenerative therapies in humans is still in its initial stages. Future research should optimize MSC application protocols by combining techniques, such as the use of nanomedicine and 3D printing for tissue engineering. Clinical studies should also understand the long-term effects and compare MSC therapies with current treatment modalities. By addressing these areas, the scientific community can ensure that MSC therapies are both safe and effective, ultimately enhancing therapeutic strategies and treatment outcomes in Periodontology and Implantology.

## Resumo

A necessidade de regenerar tecidos peri-implantares e periodontais é cada vez mais evidente. As doenças periodontais podem resultar em uma perda significativa do nível de fixação clínica, e a regeneração tecidual se apresenta como o objetivo final da terapia periodontal. Com o surgimento da osseointegração, a reabilitação protética de dentes ausentes usando implantes dentários tem aumentado, levando a uma necessidade frequente de regeneração do osso alveolar ao redor dos implantes. Esta revisão busca avaliar diversas fontes de células-tronco mesenquimais e seu potencial na regeneração de tecidos ósseos periodontais e peri-implantares. Foi realizada uma busca em sete bases de dados ao longo da última década. Três autores avaliaram de forma independente todos os títulos e resumos identificados quanto à elegibilidade, gerando tabelas para resumir estudos incluídos em animais e humanos separadamente. Um total de 55 artigos foram escolhidos para avaliação final, demonstrando cinco origens de células-tronco mesenquimais usadas em humanos e animais para regenerar tecidos periodontais e osso peri-implantar, utilizando diferentes tipos de suportes. Pesquisas das últimas décadas demonstram a eficácia das células-tronco mesenquimais na promoção da regeneração periodontal e peri-implantar. No entanto, o impacto das células-tronco mesenquimais nas terapias regenerativas em humanos ainda está em seus estágios iniciais. Pesquisas futuras devem otimizar os protocolos de aplicação das células-tronco mesenquimais combinando técnicas, como o uso de nanomedicina e impressão 3D para engenharia tecidual. Estudos clínicos também devem compreender os efeitos a longo prazo e comparar as terapias com células-tronco mesenquimais com as modalidades de tratamento atuais. Ao abordar essas áreas, a comunidade científica pode garantir que as terapias com células-tronco mesenquimais sejam seguras e eficazes, aprimorando as estratégias terapêuticas em Periodontia e Implantodontia.

**Figure:**
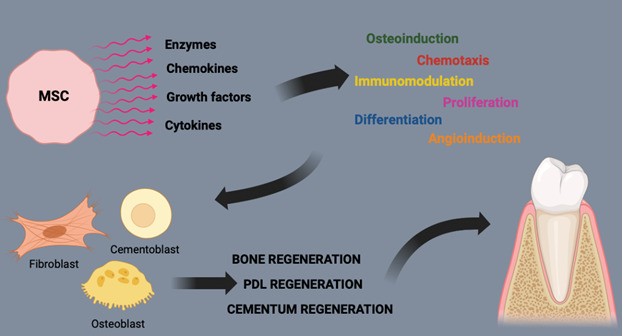

## Introduction

Following non-surgical or conventional periodontal surgical therapy, is usual to have a persistent residual deep pocket depth in sites where deep intrabony or furcation defects exist. Periodontal ressective surgery can eliminate these deep defects, however it is associated with substantial loss of clinical attachment ^1^.

Human histological studies ^2^ ^,^ ^3^ have shown that after conventional periodontal therapy, healing is characterized by the formation of a long epithelial attachment along the root surface, often with limited periodontal regeneration, mostly in the apical part of the defect.

However, over the years, new biologically guided surgical approaches and biomaterials used in periodontal regeneration therapy, have contributed to expanding its indications ^1^. In this context, periodontal regeneration implies that CAL (clinical attachment level) gain is achieved by the formation of a functional new cementum on the previously affected root surface, in conjunction with new alveolar bone and periodontal ligament ^1^ ^,^ ^4^.

Dental implant therapy has become an integral part of clinical dentistry for rehabilitating patients who lost teeth due to periodontal disease or another factor. However, it is not unusual to face situations where horizontal or vertical bone deficiencies of the alveolar process are present and may jeopardize the results for implant success and survival ^5^.

When implants are placed and a bone defect results, exposing part of the endosseous surface of the implant, the literature indicates that guided bone regeneration is a successful technique for predictable bone formation around implants ^5^ ^,^ ^6^ ^,^ ^7^. In such clinical scenarios, the combination of a membrane and bone graft or bone substitute is generally recommended for guided bone regeneration procedures to provide adequate support and enhance bone ingrowth into the defect ^5^ ^,^ ^7^. To avoid or decrease the morbidity associated with the harvesting of autogenous bone graft, the use of bone substitute materials is therefore recommended for bone regeneration at exposed implant surfaces to treat dehiscence bone defects simultaneously with implant placement ^6^ ^,^ ^7^

Current research in regenerative therapy aims to develop effective strategies to enhance the body’s ability to regenerate lost tissues, improve treatment predictability, and, at the same time, reduce surgical morbidity. In this context, efforts are focusing on growth and differentiation factors and their delivery systems, trying to identify bioactive molecules that can regulate wound and tissue regeneration, and stimulate tissue growth in the area to be regenerated around teeth and implants ^7^.

Tissue engineering relies on three main parameters: the type of cells that can be used, the scaffolds where the cells should be seeded, and the growth factor/molecular signals that should be applied ^8^. In this context, different sources of mesenchymal stromal cells (MSC) have been used to make alveolar bone tissue engineering and periodontal tissue engineering. Studies have reported significant periodontal regeneration using MSC derived from bone marrow, dental pulp, or periodontal ligament ^8^ ^,^ ^9^ ^,^ ^10^ ^,^ ^11^. Gingival fibroblasts have potentials that could be used to increase cellularity and regenerative potential since they seem to meet the requirements of MSC ^8^ ^,^ ^12^ ^,^ ^13^.

When treating intra-bony periodontal defects, studies using mesenchymal stem cells have demonstrated significant reduction in pocket depth and CAL gains, suggesting an interesting outcome in these situations ^13^ ^,^ ^14^

Therefore, taking into consideration these promising results reported in previous studies, this review aims to summarize the potential of MSC cells for use in the regeneration of bone around implants and periodontal regeneration previously described in the literature.

## Materials and methods

This scoping review was conducted following the recommendations of the Preferred Reporting Items for Systematic Reviews and Meta-Analyses - Extension for Scoping Reviews (PRISMA-ScR).

A PICO search strategy was used to define the question of this review: Population: “in vivo” (human and animal), Intervention: Mesenchymal cells for periodontal and peri-implant osteogenesis, Comparison: Type of osteogenic mesenchymal cell for periodontal and peri-implant regeneration, Outcomes: Types of mesenchymal cells present that may lead to better periodontal and peri-implant regeneration.

The search strategy was applied to seven databases PubMed/MEDLINE, Scopus, Cochrane Central Register of Controlled Trials, Embase, LILACS, ClinicalTrials.gov, and Web of Science, with a time interval of the last ten years, without language restriction, and gray literature was not included. Three separate keywords were used for the search: (stem cells) AND (peri-implant regeneration), (stem cells) AND (intrabony defect), (stem cells) AND (furcation) for each database. Inclusion and exclusion criteria for the review were determined before the reading of titles, abstracts, and full texts. Inclusion criteria: clinical trials, randomized controlled trials, control cases, case series, search time of ten years. Exclusion criteria: in vitro studies, review, systematic reviews.

The manuscripts were then uploaded to the Rayyan website (https://rayyan.qcri.org/welcome). All duplicates of the manuscripts were found and deleted using the Rayyan website tool. The application of the inclusion and exclusion criteria for each article was initially carried out by three blind researchers (KC, EK, GV) through screening of titles and abstracts. Conflicts were resolved by discussion between the three reviewers as well as the eligibility of the analysis.

## Results

**Figure 1:**
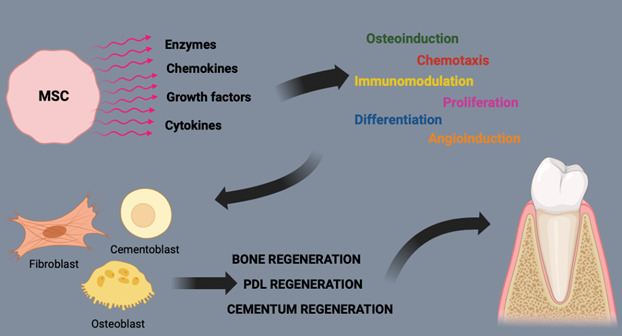
Study flowchart.

A total of 788 abstracts (167 in Pudmed/MEDLINE, 30 in Scopus, 41 in the Cochrane Central Register of Controls, 4 in LILACS, 2 in Clinical Trials.gov, 202 in Embase, and 342 in Web of Science) between 2004 and 2022 have been retrieved from the systematic search. Excluding duplicate manuscripts, totaling 480 abstracts to be reviewed. The titles and abstracts were independently reviewed by three reviewers to determine which of the 480 articles would proceed to full-text analysis, within the predetermined inclusion and exclusion criteria in the search project. Following this, 417 manuscripts were removed for not meeting the inclusion criteria or being outside the scope of the research. Concluding this phase, 63 abstracts were selected for full-text analysis. Of these 63 manuscripts, 48 were animal studies and 15 were human studies. Following the complete reading, 7 of the 63 manuscripts were excluded, leaving a final set of 55 articles, 42 in animals and 13 in humans, to comprise the final set of manuscripts included in this scoping review. A workflow diagram was created to visualize the work steps from identification, and screening to final inclusion (Figure 1).

Tables were created separating these two types of studies (animal and human). In the animal studies table, authors, country, cell origin, scaffold type, bone defect type, human pathology, evaluated methodology, follow-up time, and results were listed. In the human studies table, authors, country, cell origin, study type, sample size, defect type, evaluation method, follow-up time, and results were listed (Box 1 and Box 2).

## Studies Using Animal Models

### Animal Models and Type of Bone Defects

**Figure 2:**
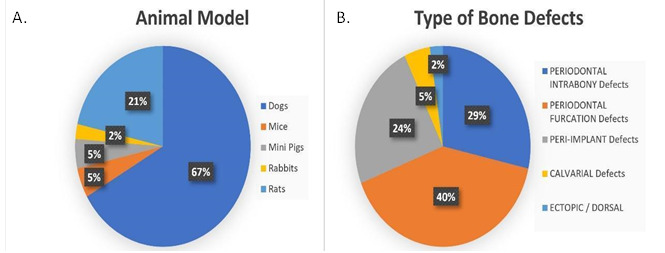
(a) Animal Model and (b) Type of Bone Defect.

Dogs were the animal model most used to quantify bone defect repair, the experimental subjects were treated with scaffolds associated with some types of stromal cells (28 out of 42 experiments) ^18^ ^,^ ^19^ ^,^ ^20^ ^,^ ^21^ ^,^ ^22^ ^,^ ^23^ ^,^ ^24^ ^,^ ^25^ ^,^ ^26^ ^,^ ^27^ ^,^ ^28^ ^,^ ^29^ ^,^ ^30^ ^,^ ^31^ ^,^ ^32^ ^,^ ^33^ ^,^ ^34^ ^,^ ^35^ ^,^ ^36^ ^,^ ^37^ ^,^ ^38^ ^,^ ^39^ ^,^ ^40^ ^,^ ^41^ ^,^ ^42^ ^,^ ^43^ ^,^ ^44^ ^,^ ^45^. Rats were used in 9 out of 42 experiments ^46^ ^,^ ^47^ ^,^ ^48^ ^,^ ^49^ ^,^ ^50^ ^,^ ^51^ ^,^ ^52^ ^,^ ^53^ ^,^ ^54^, while Mice and Minipigs represent 10% (5% each) ^55^ ^,^ ^56^ ^,^ ^57^ ^,^ ^58^ of the total amount of manuscripts and only 2% of the manuscripts used Rabbits as animal models ^59^. The types of bone defects could not be compared among the experiments, because of the different methods that were applied to build them, but, out of 42 experiments, 17 were simulating periodontal furcation defects ^19^ ^,^ ^24^ ^,^ ^25^ ^,^ ^26^ ^,^ ^27^ ^,^ ^28^ ^,^ ^29^ ^,^ ^30^ ^,^ ^31^ ^,^ ^32^ ^,^ ^33^ ^,^ ^34^ ^,^ ^35^ ^,^ ^36^ ^,^ ^39^ ^,^ ^50^ ^,^ ^58^, 12 periodontal ^23^ ^,^ ^37^ ^,^ ^41^ ^,^ ^42^ ^,^ ^43^ ^,^ ^45^ ^,^ ^46^ ^,^ ^48^ ^,^ ^49^ ^,^ ^51^ ^,^ ^54^ and 10 peri-implant defects ^18^ ^,^ ^20^ ^,^ ^21^ ^,^ ^22^ ^,^ ^38^ ^,^ ^40^ ^,^ ^44^ ^,^ ^52^ ^,^ ^57^ ^,^ ^59^. Also aiming for oral bone repair 2 authors described calvarial defects ^47^ ^,^ ^55^ and 1 showed an ectopic/dorsal site ^56^. These findings may reflect that the jaws are the main site to represent a periodontal or peri-implant defect. These results can be observed in Figure 2.

### The Human Pathology

**Figure 3:**
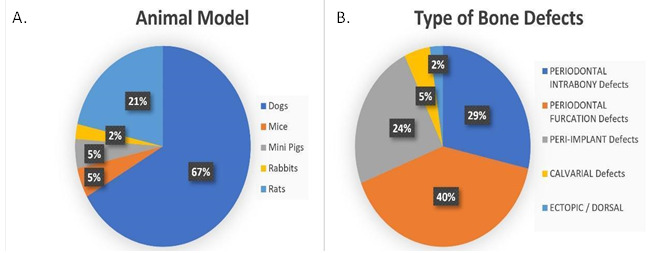
Human Pathology.

Most of the objectives of the studies were focused on Inflammatory Periodontal Disease, 33 out of 42 ^19^ ^,^ ^20^ ^,^ ^23^ ^,^ ^24^ ^,^ ^25^ ^,^ ^26^ ^,^ ^27^ ^,^ ^28^ ^,^ ^29^ ^,^ ^30^ ^,^ ^31^ ^,^ ^32^ ^,^ ^33^ ^,^ ^34^ ^,^ ^35^ ^,^ ^36^ ^,^ ^37^ ^,^ ^39^ ^,^ ^41^ ^,^ ^42^ ^,^ ^43^ ^,^ ^44^ ^,^ ^45^ ^,^ ^46^ ^,^ ^47^ ^,^ ^48^ ^,^ ^49^ ^,^ ^50^ ^,^ ^51^ ^,^ ^52^ ^,^ ^53^ ^,^ ^54^ ^,^ ^55^ ^,^ ^56^. Inflammatory Peri-Implant Disease was the second porpoise most studied found in this review, 8 out of 42 ^18^ ^,^ ^21^ ^,^ ^22^ ^,^ ^38^ ^,^ ^40^ ^,^ ^44^ ^,^ ^52^ ^,^ ^54^ ^,^ ^59^. One study was engaged to answer for oral bone with no specific site ^47^ (Figure 3).

### Stromal Cell Origin

**Figure 4:**
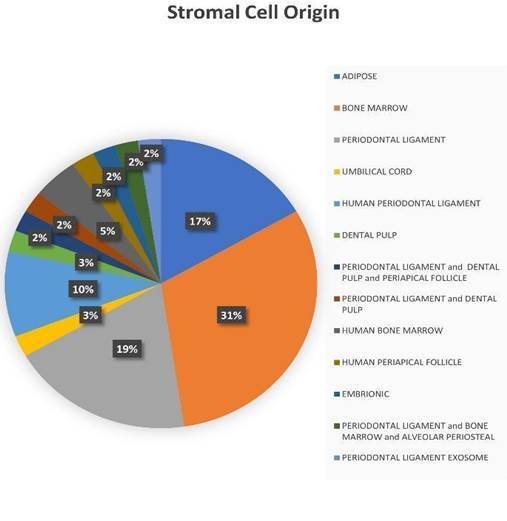
Stromal Cell Origin.

The main finding of this review was the type of Stromal Cell Origin that leads the periodontal and peri-implant research. The most used Stromal Cell Origin was the bone marrow stromal cells (31%) ^21^ ^,^ ^24^ ^,^ ^27^ ^,^ ^28^ ^,^ ^29^ ^,^ ^32^ ^,^ ^33^ ^,^ ^34^ ^,^ ^36^ ^,^ ^37^ ^,^ ^38^ ^,^ ^39^ ^,^ ^44^ ^,^ ^45^ ^,^ ^52^ ^,^ ^60^ ^,^ ^61^ ^,^ ^62^ followed by a periodontal ligament (19%) ^23^ ^,^ ^30^ ^,^ ^31^ ^,^ ^35^ ^,^ ^37^ ^,^ ^41^ ^,^ ^42^ ^,^ ^43^ ^,^ ^53^ ^,^ ^54^ ^,^ ^63^ ^,^ ^64^ ^,^ ^65^ ^,^ ^66^ ^,^ ^67^ ^)^ and adipose origin (17%) ^20^ ^,^ ^26^ ^,^ ^46^ ^,^ ^51^ ^,^ ^52^ ^,^ ^59^ A representative percentage of 10% of human periodontal ligament stromal cells ^19^ ^,^ ^49^ ^,^ ^55^ ^,^ ^56^ and 5% ^46^ ^,^ ^60^ ^,^ ^61^ human bone marrow, shows the heterologous part of the intention of the authors in using this way of treatment with stromal cells 3% were derived from umbilical cord ^22^, 2 % of the manuscripts described the dental pulp stromal cells for their experiments ^10^ ^,^ ^25^ ^,^ ^30^ ^,^ ^41^ ^,^ ^68^ ^,^ ^69^ ^,^ ^70^ and the others divided an equal percentage of 1% of the total number animal experiments ^30^ ^,^ ^58^ ^,^ ^71^, as shown in Figure 4.

### Method of evaluation

The evaluations in most of the manuscripts were conducted with micro-CT and histological analysis methods, such as histomorphometry and Immunohistochemistry had an important place in the methods of evaluation for many articles. All methods of evaluation were employed to compare and establish significant results for in vivo periodontal/peri-implant defect repair using stromal cells associated with some type of scaffold (Box 1).

**Box 1:**
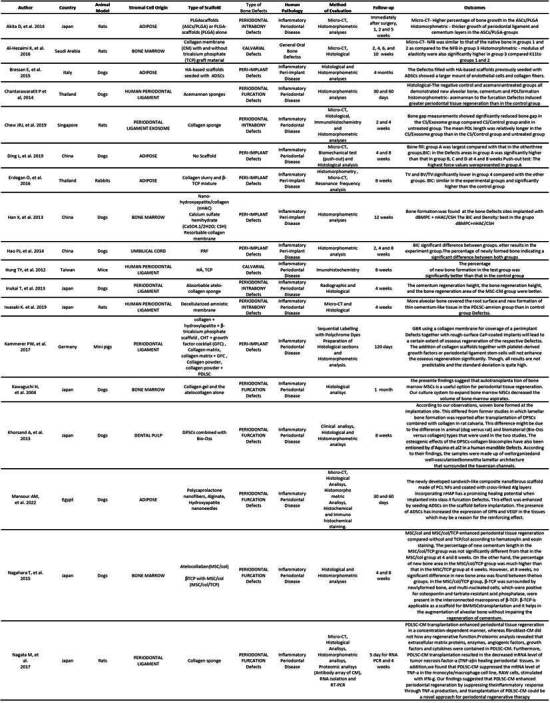
Characteristics of the studies in animal models.

**Box 1:**
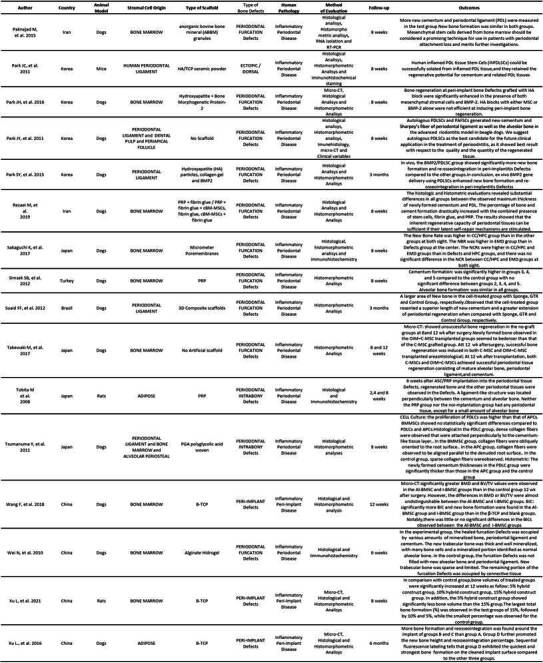
Continuation

**Box 1:**
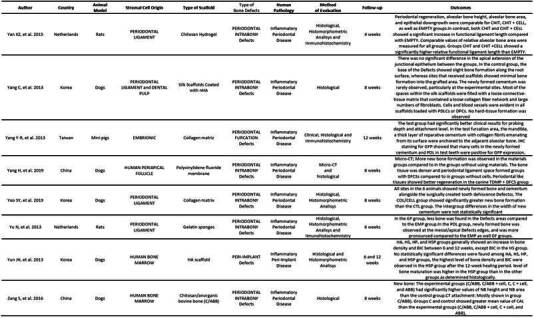
Continuation

**Box 2:**
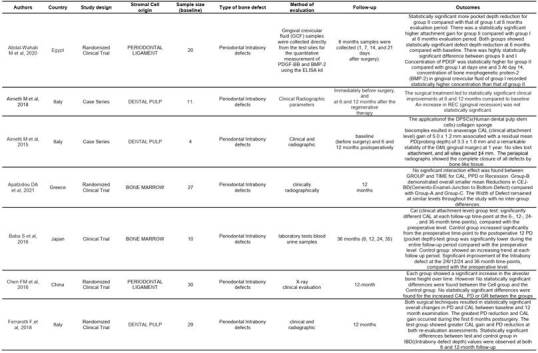
Characteristics of the studies in humans.

CAL: Clinical Attachment Level)

PD: Probe Depth

**Box 2:**
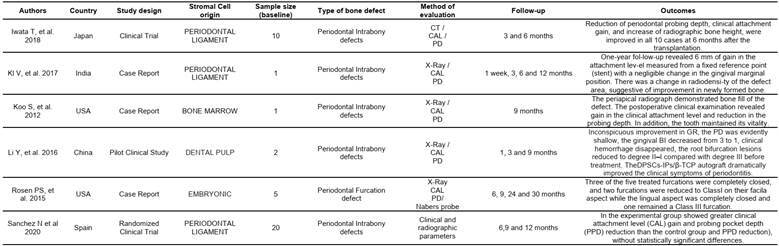
Continuation

## Clinical Studies

### Study Design

**Figure 5:**
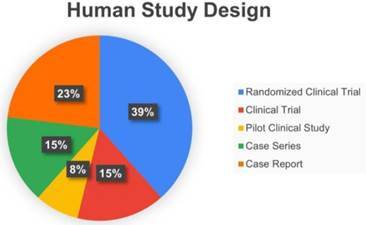
Human Study Design.

All manuscripts included as a Clinical Study were counted in a total of 13. This scope review described the design of studies as: Randomized Clinical Trials, representing 5 of the total manuscripts ^60^ ^,^ ^63^ ^,^ ^64^ ^,^ ^67^ ^,^ ^69^; Case Reports, representing 3 manuscripts ^62^ ^,^ ^66^ ^,^ ^71^; Clinical trials, representing 2 manuscripts ^61^ ^,^ ^65^; Case Series, representing 2 Manuscripts ^10^ ^,^ ^68^ and 1 Pilot Clinical Study ^70^ (Figure 5).

### Type of Bone Defect

**Figure 6:**
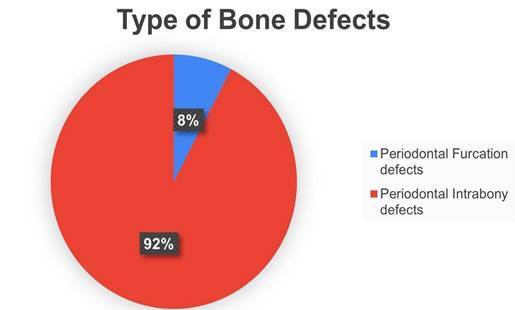
Type of Bone Defect.

The bone defects created for the experiments were periodontal in total, but almost all of them were treating periodontal intrabony defects ^60^ ^,^ ^61^ ^,^ ^62^ ^,^ ^63^ ^,^ ^64^ ^,^ ^65^ ^,^ ^66^ ^,^ ^67^ ^,^ ^68^ ^,^ ^69^ ^,^ ^70^ ^,^ ^10^ during the clinical manuscripts. Only one out of 13 was described as treating a periodontal furcation defect ^71^ (Figure 6).

### Stromal Cell Origin

**Figure 7:**
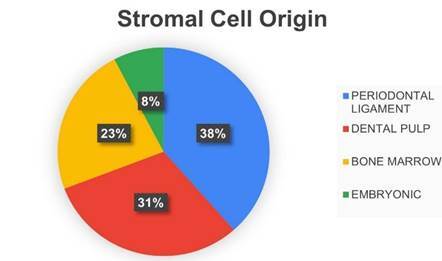
Stromal Cell Origin.

Regarding the origin of the stromal cell used in the techniques described for clinical treatments the most used stromal cells were the periodontal ligament ^5^ ^) (^ ^63^ ^,^ ^64^ ^,^ ^65^ ^,^ ^66^ ^,^ ^67^, followed by dental pulp ^4^ ^) (^ ^10^ ^,^ ^68^ ^,^ ^69^ ^,^ ^70^, bone marrow ^3^ ^) (^ ^60^ ^,^ ^61^ ^,^ ^62^ and finally by embryonic stromal cells ^70^ (Figure 7).

### Outcomes

All manuscripts included in this scoping review showed the results during bone periodontal/peri-implant repair using any kind of stromal cell were listed with other information and their outcomes in Box 1(Animals) and observations Box 2(Human).

## Discussion

This scoping review compiled the use of animal models in the study of mesenchymal stem cells (MSCs) for tissue engineering in the treatment of peri-implant alterations, intraosseous defects, and periodontal diseases, in addition to reporting the use of stem cells in humans in periodontics. The success of periodontal regeneration via tissue engineering depends on factors such as a supply of progenitor cells capable of differentiating into specific phenotypes, appropriate signals for cellular differentiation, and a supporting structure. MSCs are prime candidates due to their ability to differentiate into various cell types (Graphic Abstract). MSCs must be capable of differentiating into osteogenic, chondrogenic, and adipogenic cells, in addition to their immunosuppressive properties ^72^. In periodontitis, MSCs can modulate the immune response, reducing inflammation and promoting healing ^73^. However, the constant exposure of periodontal tissues to a complex microbiota creates a continuous inflammatory environment that can affect the function of periodontal ligament stem cells but can also activate mechanisms of resolution and return to homeostasis ^74^.

The transition from the inflammatory phase to the proliferative phase is crucial for tissue healing and regeneration, involving the recruitment of fibroblasts from the wound edges, and the epithelial-mesenchymal transition involves the formation of epithelium from keratinocytes. Periodontal ligament stem cells (PDL) are multipotent progenitor cells and, therefore, capable of proliferating and differentiating into the various cell types necessary for the periodontium, indicating that periodontal tissues can regenerate if these cells can be mobilized ^75^.

In peri-implantitis, the ability of MSCs to modulate the immune response can reduce inflammation around the implant and promote tissue regeneration, in addition to forming new bone and blood vessels, ensuring their survival and proper integration with existing periodontal structures ^76^ ^,^ ^77^ ^,^ ^78^. MSCs can be combined with biomaterials to enhance their regenerative potential, as these can serve as structural support and improve the delivery and efficacy of MSCs at the treatment site, as demonstrated by the different types of scaffolds used for the treatment of periodontal and peri-implant diseases in the research ^79^. Different animal models, such as rats, dogs, pigs, and mice, are used to study tissue engineering with MSCs, each offering distinct advantages. This review observed 28 studies with dog models ^18^ ^,^ ^19^ ^,^ ^20^ ^,^ ^21^ ^,^ ^22^ ^,^ ^23^ ^,^ ^24^ ^,^ ^25^ ^,^ ^26^ ^,^ ^27^ ^,^ ^28^ ^,^ ^29^ ^,^ ^30^ ^,^ ^31^ ^,^ ^32^ ^,^ ^33^ ^,^ ^34^ ^,^ ^35^ ^,^ ^36^ ^,^ ^37^ ^,^ ^38^ ^,^ ^39^ ^,^ ^40^ ^,^ ^41^ ^,^ ^42^ ^,^ ^43^ ^,^ ^44^ ^,^ ^45^, 9 with rats ^46^ ^,^ ^47^ ^,^ ^48^ ^,^ ^49^ ^,^ ^50^ ^,^ ^51^ ^,^ ^52^ ^,^ ^53^ ^,^ ^54^, 2 with mice ^55^ ^,^ ^56^, 1 with rabbits ^59^, and 2 with minipigs ^59^.

Studies using canine models are predominant due to their anatomical and physiological similarities of periodontal tissues with humans, including factors such as bone density, cell renewal rate, and healing processes ^18^ ^,^ ^57^. The spacious oral cavity of dogs facilitates manipulation and creation of significant periodontal defects, allowing the use of scaffolds of different sizes and shapes ^25^ ^,^ ^26^. Their immune system, like humans, permits comparative observation of the effects of mesenchymal stem cells ^19^ ^,^ ^31^. The long lifespan of dogs allows for long-term effect studies on periodontal regeneration ^20^ ^,^ ^21^. The canine oral microbiota, although not identical, is more similar to that of humans, influencing regenerative treatments ^29^ ^,^ ^41^. The progression of periodontal disease in dogs is comparable to that in humans, making them ideal models for studies and therapeutic interventions ^36^ ^,^ ^59^. Despite requiring more resources, canine models provide an approach that is closer to human conditions, allowing for more predictive extrapolation of research results to potential clinical applications.

The MSCs used in tissue engineering can be obtained from various tissues, including bone marrow, adipose tissue, dental pulp, periodontal ligament, and umbilical cord among others. The choice of MSC source is critical, as it can influence their differentiation capabilities, immunomodulatory properties, and overall therapeutic potential. This review has recorded the use of MSCs from periodontal ligaments in 17 studies ^19^ ^,^ ^23^ ^,^ ^30^ ^,^ ^31^ ^,^ ^35^ ^,^ ^37^ ^,^ ^41^ ^,^ ^42^ ^,^ ^43^ ^,^ ^44^ ^,^ ^48^ ^,^ ^49^ ^,^ ^50^ ^,^ ^55^ ^,^ ^56^ ^,^ ^57^, bone marrow in 17 studies ^21^ ^,^ ^24^ ^,^ ^27^ ^,^ ^28^ ^,^ ^29^ ^,^ ^32^ ^,^ ^33^ ^,^ ^34^ ^,^ ^36^ ^,^ ^37^ ^,^ ^38^ ^,^ ^39^ ^,^ ^44^ ^,^ ^45^ ^,^ ^52^ ^,^ ^60^ ^,^ ^61^ ^,^ ^62^, adipose tissue in 7 studies ^18^ ^,^ ^20^ ^,^ ^26^ ^,^ ^46^ ^,^ ^51^ ^,^ ^52^ ^,^ ^59^, umbilical cords in one study ^22^, dental pulp in tree study ^10^ ^,^ ^25^ ^,^ ^30^ ^,^ ^41^ ^,^ ^68^ ^,^ ^69^ ^,^ ^70^, and one study on embryonic stem cells ^58^. These selections often reflect a balance between the high regenerative potential of the cells and practical considerations such as cell availability and the invasiveness of the harvesting procedure.

The study by Pinheiro et al . ^2019^ highlighted that mesenchymal stem cells (MSCs) can be obtained less invasively from sites such as the umbilical cord, the orbicularis oris muscle, and deciduous dental pulp. These cells exhibit excellent osteogenic potential due to their neural crest characteristics, which predispose them to greater osteogenic differentiation. The use of dental pulp stem cells has shown satisfactory clinical results in the bone consolidation of cleft lip and palate, with benefits such as shorter operative time, reduced postoperative pain, costs, and hospitalization time. The results indicate that regeneration with this type of cell can be promising ^81^.

This scoping review highlights the efficacy of MSCs obtained from the periodontal ligament, bone marrow, and adipose tissue, which have been more largely used and validated in the literature reviewed for treating periodontal disease ^19^ ^,^ ^23^ ^,^ ^30^ ^,^ ^31^ ^,^ ^35^ ^,^ ^37^ ^,^ ^41^ ^,^ ^42^ ^,^ ^43^ ^,^ ^44^ ^,^ ^48^ ^,^ ^49^ ^,^ ^50^ ^,^ ^55^ ^,^ ^56^ ^,^ ^57^ ^,^ ^21^ ^,^ ^24^ ^,^ ^27^ ^,^ ^28^ ^,^ ^29^ ^,^ ^32^ ^,^ ^33^ ^,^ ^34^ ^,^ ^36^ ^,^ ^37^ ^,^ ^38^ ^,^ ^39^ ^,^ ^44^ ^,^ ^45^ ^,^ ^52^ ^,^ ^60^ ^,^ ^61^ ^,^ ^62^ ^,^ ^20^ ^,^ ^26^ ^,^ ^46^ ^,^ ^51^ ^,^ ^52^ ^,^ ^59^.

The PDLSCs are especially suited for this role, given their involvement in the maintenance and repair of periodontal tissues and they can be harvested from extracted teeth, offering a source of cells without the need for additional invasive procedures. These PDLSCs have shown the potential to differentiate into various cell types that constitute the periodontium, which is vital for periodontal regeneration ^19^ ^,^ ^23^ ^,^ ^30^ ^,^ ^31^ ^,^ ^35^ ^,^ ^37^ ^,^ ^41^ ^,^ ^42^ ^,^ ^43^ ^,^ ^44^ ^,^ ^48^ ^,^ ^49^ ^,^ ^50^ ^,^ ^55^ ^,^ ^56^ ^,^ ^57^ ^,^ ^63^ ^,^ ^64^ ^,^ ^65^ ^,^ ^66^ ^,^ ^67^. Bone marrow-derived stem cells (BMSCs) are recognized for their high differentiation potential, supporting the formation of bone, cartilage, and a functional periodontal ligament, in addition to their ability to modulate the immune response, an advantage in controlling associated inflammation. to periodontal diseases ^21^ ^,^ ^24^ ^,^ ^27^ ^,^ ^28^ ^,^ ^29^ ^,^ ^32^ ^,^ ^33^ ^,^ ^34^ ^,^ ^36^ ^,^ ^37^ ^,^ ^38^ ^,^ ^39^ ^,^ ^44^ ^,^ ^45^ ^,^ ^52^ ^,^ ^60^ ^,^ ^61^ ^,^ ^62^. The third most used cell source of MSC was the adipose-derived stem cells (ADSCs). They can be isolated in large quantities from adipose tissue and have been demonstrated to differentiate into cell types crucial for periodontal regeneration, such as osteogenic lineage ^18^ ^,^ ^20^ ^,^ ^26^ ^,^ ^46^ ^,^ ^51^ ^,^ ^52^ ^,^ ^59^.

The review also showed MSC sources referenced (PLSC, BMSC, ADSC) are capable of secreting bioactive factors that modulate the inflammatory environment and recruit other progenitor cells ^19^ ^,^ ^23^ ^,^ ^30^ ^,^ ^31^ ^,^ ^35^ ^,^ ^37^ ^,^ ^4^ ^,^ ^42^ ^,^ ^43^ ^,^ ^44^ ^,^ ^48^ ^,^ ^49^ ^,^ ^50^ ^,^ ^55^ ^,^ ^46^ ^,^ ^57^ ^,^ ^63^ ^,^ ^64^ ^,^ ^65^ ^,^ ^66^ ^,^ ^67^ ^,^ ^21^ ^,^ ^24^ ^,^ ^27^ ^,^ ^28^ ^,^ ^29^ ^,^ ^32^ ^,^ ^33^ ^,^ ^34^ ^,^ ^36^ ^,^ ^67^ ^,^ ^68^ ^,^ ^39^ ^,^ ^44^ ^,^ ^45^ ^,^ ^52^ ^,^ ^60^ ^,^ ^61^ ^,^ ^62^ ^,^ ^18^ ^,^ ^20^ ^,^ ^26^ ^,^ ^46^ ^,^ ^51^ ^,^ ^52^ ^,^ ^59^, but these characteristics are inherent to all MSCs and have been detailed by authors who also utilized other sources like umbilical cord, dental pulp, and embryonic stem cells to make the treatment of periodontal disease in animal model studies and got success in their results ^22^ ^,^ ^10^ ^,^ ^25^ ^,^ ^30^ ^,^ ^41^ ^,^ ^68^ ^,^ ^69^ ^,^ ^70^ ^,^ ^58^.

A single article from this review focused on the regeneration of intraosseous periodontal defects in rats using exosomes embedded in a collagen matrix, vesicles that replicate the therapeutic capacity of mesenchymal cells in the studied animal model ^48^. Significant bone and periodontal tissue regeneration can be observed, indicating improved migration, proliferation, matrix synthesis, and differentiation into new bone as well as restoration of the periodontal ligament. This demonstrates a promising path in the use of exosomes from human mesenchymal cells in periodontal regeneration.

Signaling vesicles, highlighted for their regenerative capacity, have received special attention recently. Pinheiro et al, 2023 ^82^, emphasized in a brief review how these extracellular vesicles can positively influence bone tissue regeneration, following the stages of inflammation, repair, and remodeling, and involving multiple signaling pathways in bone. A subsequent review highlighted those therapeutic exosomes, generally obtained from dental pulp, periodontal ligament cells, gingival fibroblasts, stem cells from exfoliated deciduous teeth, and the apical papilla, facilitated the regenerative potential of tissues such as bone and periodontal tissue, as well as assisting in orthodontic treatments and alleviating temporomandibular joint disorders and others. The 113 articles analyzed confirmed the efficacy of exosomes in 100% of the cases, indicating that, in bone regeneration, they are as effective or more effective than rhBMP2, with the additional advantage of reducing inflammation. This finding underscores the still unexplored potential of exosomes in oral regeneration ^83^.

The importance of scaffolds in tissue engineering, especially in tissue regeneration mediated by mesenchymal stem cells, should be emphasized. Scaffolds are critical for tissue engineering and can be made from materials such as natural and synthetic polymers, hydrogels, and ceramics with distinct properties. They are essential for providing support for the adhesion, proliferation, and differentiation of MSCs. The choice of scaffold material is crucial, as its specific properties directly influence the success of tissue regeneration. This review evaluates successful scaffolds, guiding future selections for periodontal regeneration (Box 1). Overall, the reviewed studies demonstrate that the combination of MSCs with scaffolds leads to better tissue regeneration compared to the use of scaffolds alone for the treatment of periodontal disease ^46^, and the choice of scaffold type can influence the mechanical properties of the regenerated tissue ^47^.

This review's findings are instrumental for selecting the appropriate animal model, MSC source, and scaffold material for further periodontal tissue engineering research. The comparison of various evaluation methods across studies, such as micro-CT scans and histomorphometric analysis, contributes to standardizing outcome measures in this field and all this data can be observed in Box 1.

When human studies were evaluated for this scoping review, a diverse range of study designs, cell origins, and methodological assessments were observed across different countries, highlighting the global effort in utilizing MSCs for periodontal repair ^10^ ^,^ ^60^ ^,^ ^62^ ^,^ ^63^ ^,^ ^64^ ^,^ ^65^ ^,^ ^66^ ^,^ ^67^ ^,^ ^68^ ^,^ ^69^ ^,^ ^70^ ^,^ ^71^.

The clinical application of MSCs in periodontal therapy described in this manuscript explored promising outcomes across various study designs and evaluation methods. A significant contribution to this field was made by four authors who conducted a randomized clinical trial ^60^ ^,^ ^63^ ^,^ ^64^ ^,^ ^67^ ^,^ ^69^. Other studies such as case series, cohort studies, and clinical case reports have also been described ^62^ ^,^ ^66^ ^,^ ^71^ ^,^ ^61^ ^,^ ^65^ ^,^ ^10^ ^,^ ^68^ ^,^ ^70^ opening new avenues for the use of tissue engineering strategies using MSCs and scaffolds to regenerate tissues affected by periodontal disease. While there is a generally positive trend towards using MSCs, varying degrees of clinical success were observed in this scoping review it is a necessity for further standardized long-term studies development to confirm their therapeutic efficacy fully.

Promising results have been described in this manuscript for the clinical application of MSCs with various study designs and evaluation methods such as randomized clinical trials ^60^ ^,^ ^63^ ^,^ ^64^ ^,^ ^67^ ^,^ ^69^, case series, cohort studies, and clinical case reports ^62^ ^,^ ^66^ ^,^ ^71^ ^,^ ^61^ ^,^ ^65^ ^,^ ^10^ ^,^ ^68^ ^,^ ^70^, opening new avenues for the use of MSCs and scaffolds to regenerate periodontal tissues. However, the development of more standardized long-term studies is necessary to confirm the therapeutic efficacy.

When comparing studies on periodontal regeneration using animal models and those conducted in humans, several key differences emerge. Animal models, often using rodents, dogs, or non-human primates, provide a controlled environment where variables can be tightly regulated, allowing for a detailed examination of cellular and molecular mechanisms involved in periodontal regeneration. These models enable invasive procedures and frequent sampling that are not feasible in human studies, facilitating a deeper understanding of tissue responses and healing processes. However, the anatomical, physiological, and host-microbe interaction differences between animal models and humans can limit the direct applicability of findings to clinical practice. Human studies, on the other hand, offer the advantage of directly assessing the efficacy and safety of regenerative therapies in the target population. These studies are critical for evaluating clinical outcomes, patient-related factors, and long-term benefits of treatment, yet they often face ethical constraints, variability in patient conditions, and limited opportunities for invasive monitoring. However, clinical trials are costly and time-consuming. Moreover, human studies involving MSCs encounter numerous regulatory challenges to be implemented. Thus, while animal models are invaluable for foundational research and mechanistic insights, human studies are essential for translating these findings into effective clinical treatments.

Research from the past decades supports the effectiveness of MSC in promoting periodontal and peri-implant regeneration. However, the impact of MSC on regenerative therapies in humans is still in its initial steps. Many existing studies are preclinical, some are in early trial stages, and a few are well-designed longitudinal clinical trials. Future research should optimize MSC application protocols by combining techniques, such as the use of nanomedicine and 3D printing for tissue engineering. Clinical studies should also understand the long-term effects and compare MSC therapies with current treatment modalities. By addressing these areas, the scientific community can ensure that MSC therapies are both safe and effective, ultimately enhancing therapeutic strategies and treatment outcomes in Periodontology and Implantology.
